# *catena*-Poly[[[aqua­(5-carb­oxy­pyridine-3-carboxyl­ato-κ*N*)copper(I)]-μ-4,4′-bipyridine-κ^2^ *N*:*N*′] monohydrate]

**DOI:** 10.1107/S1600536811055656

**Published:** 2012-01-07

**Authors:** Gang Liu, Gao Qin

**Affiliations:** a College of Food Engineering, Jilin Teachers’ Institute of Engineering and Technology, 130052 Changchun, Jilin, People’s Republic of China

## Abstract

In the title compound, {[Cu(C_7_H_4_NO_4_)(C_10_H_8_N_2_)(H_2_O)]·H_2_O}_*n*_, the Cu^I^ ion is coordinated by the N atom from a 5-carb­oxy­pyridine-3-carboxyl­ate anion, two N atoms from two 4,4′-bipyridine (4,4′-bipy) ligands and one water mol­ecule in a distorted tetra­hedral geometry. The 4,4′-bipy ligands bridge the Cu^I^ ions into polymeric chains propagating in [201]. The latticeand the coordinating water mol­ecules as well as the carboxy OH function are involved in the formation of inter­molecular O—H⋯O hydrogen bonds, which consolidate the crystal packing.

## Related literature

For related structures of derivatives of pyridine-3,5-dicarb­oxy­lic acid in coordination chemistry, see: Qin *et al.* (2002 ▶); Eubank *et al.* (2007 ▶); Mirtschin *et al.* (2008 ▶); Banerjee *et al.* (2010 ▶, 2011 ▶). 
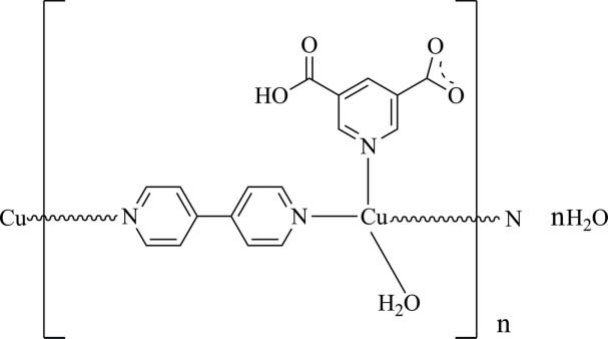


## Experimental

### 

#### Crystal data

[Cu(C_7_H_4_NO_4_)(C_10_H_8_N_2_)(H_2_O)]·H_2_O
*M* *_r_* = 421.87
Monoclinic, 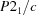

*a* = 10.6511 (13) Å
*b* = 23.321 (3) Å
*c* = 7.0111 (8) Å
β = 105.044 (7)°
*V* = 1681.9 (3) Å^3^
*Z* = 4
Mo *K*α radiation
μ = 1.34 mm^−1^
*T* = 298 K
0.30 × 0.20 × 0.12 mm

#### Data collection

Bruker SMART APEXII CCD area-detector diffractometer
Absorption correction: multi-scan (*SADABS*; Bruker, 2005 ▶) *T*_min_ = 0.689, *T*_max_ = 0.856
13086 measured reflections
2971 independent reflections
2330 reflections with *I* > 2σ(*I*)
*R*_int_ = 0.038

#### Refinement

*R*[*F* ^2^ > 2σ(*F* ^2^)] = 0.036
*wR*(*F* ^2^) = 0.127
*S* = 1.03
2971 reflections
245 parameters
H-atom parameters constrained
Δρ_max_ = 0.50 e Å^−3^
Δρ_min_ = −0.50 e Å^−3^

### 

Data collection: *APEX2* (Bruker, 2005 ▶); cell refinement: *SAINT* (Bruker, 2005 ▶); data reduction: *SAINT*; program(s) used to solve structure: *SHELXTL* (Sheldrick, 2008 ▶); program(s) used to refine structure: *SHELXTL*; molecular graphics: *SHELXTL*; software used to prepare material for publication: *SHELXTL*.

## Supplementary Material

Crystal structure: contains datablock(s) global, I. DOI: 10.1107/S1600536811055656/cv5224sup1.cif

Structure factors: contains datablock(s) I. DOI: 10.1107/S1600536811055656/cv5224Isup2.hkl

Additional supplementary materials:  crystallographic information; 3D view; checkCIF report

## Figures and Tables

**Table 1:** Hydrogen-bond geometry (Å, °)

*D*—H⋯*A*	*D*—H	H⋯*A*	*D*⋯*A*	*D*—H⋯*A*
O1—H1*A*⋯O6^i^	0.82	1.69	2.502 (3)	168
O5—H5*WB*⋯O2^ii^	0.85	2.12	2.959 (3)	167
O6—H6*WA*⋯O3^ii^	0.85	1.91	2.694 (3)	152
O5—H5*WA*⋯O3^iii^	0.85	1.98	2.809 (3)	165
O6—H6*WB*⋯O4^iv^	0.85	1.84	2.682 (3)	171

Symmetry codes: (i) 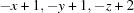; (ii) 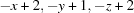; (iii) 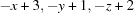; (iv) 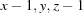.

**Fig. 1.**
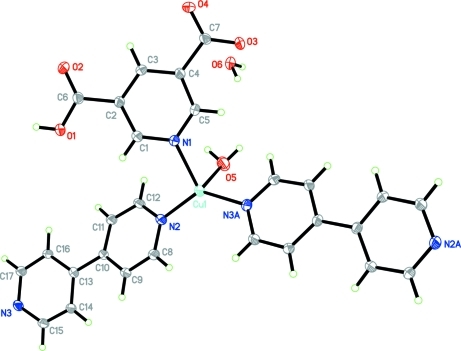
The molecular structure of (I), with displacement ellipsoids drawn at the 30% probability level [symmetry codes: (A) 1 + x, 1/2 - y, 1/2 + z].

**Fig. 2.**
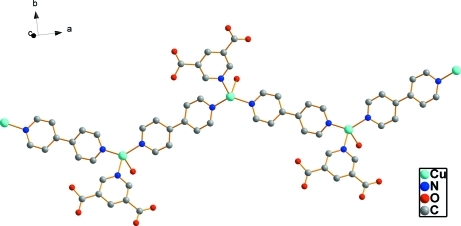
A portion of the polymeric chain in (I). H atoms have been omitted for clarity.

## supplementary crystallographic information

### Comment

The construction of metal complexes based on pyridine-3,5-dicarboxylic acid has attracted much attention (Qin *et al.*, 2002; Eubank *et al.*, 2007; Mirtschin *et al.*, 2008; Banerjee *et al.*, 2010, 2011). In our search for new metal complexes based on pyridine-3,5-dicarboxylic acid ligand, the title complex, (I), was synthesized and its crystal structure determined (Fig. 1).

In the crystal structure, the 4,4'-bipyridine ligands bridge the Cu^I^ ions into polymeric chains propagated in direction [201] (Fig. 2). Lattice water molecules are involved in formation of intermolecular O—H···O hydrogen bonds (Table 1), which consolidate the crystal packing.

### Experimental

A mixture of Cu(NO_3_)_2_^.^3H_2_O (0.10 mmol), pyridine-3,5-dicarboxylic acid (0.20 mmol), 4,4'-bipyridine (0.10 mmol) and H_2_O (3 ml) was sealed in a 10 ml Tefon-lined stainless-steel reactor and then heated to 398 K for 96 h under autogenous pressure. The mixture was slowly cooled to room temperature. Yellow block crystals suitable for X-ray diffraction analysis were collected by filtration.

### Refinement

H atoms attached to C atoms were placed in calculated positions (C—H = 0.93 Å) and refined as riding atoms, with *U*_iso_(H) = 1.2 *U*_eq_(C). The hydroxyl and water H atoms were located in a difference map, but placed in idealized positions (O—H 0.82 - 0.85 Å) and refined as riding, with *U*_iso_(H) = 1.5 *U*_eq_(O).

### Crystal data

**Table:**

[Cu(C_7_H_4_NO_4_)(C_10_H_8_N_2_)(H_2_O)]·H_2_O	*F*(000) = 864
*M**_r_* = 421.87	*D*_x_ = 1.666 Mg m^−^^3^
Monoclinic, *P*2_1_/*c*	Mo *K*α radiation, λ = 0.71073 Å
Hall symbol: -P 2ybc	Cell parameters from 4034 reflections
*a* = 10.6511 (13) Å	θ = 2.6–26.3°
*b* = 23.321 (3) Å	µ = 1.34 mm^−^^1^
*c* = 7.0111 (8) Å	*T* = 298 K
β = 105.044 (7)°	Block, yellow
*V* = 1681.9 (3) Å^3^	0.30 × 0.20 × 0.12 mm
*Z* = 4	

### Data collection

**Table:**

Bruker SMART APEXII CCD area-detector diffractometer	2971 independent reflections
Radiation source: fine-focus sealed tube	2330 reflections with *I* > 2σ(*I*)
graphite	*R*_int_ = 0.038
φ and ω scans	θ_max_ = 25.0°, θ_min_ = 1.8°
Absorption correction: multi-scan (*SADABS*; Bruker, 2005)	*h* = −12→11
*T*_min_ = 0.689, *T*_max_ = 0.856	*k* = −27→27
13086 measured reflections	*l* = −8→8

### Refinement

**Table:**

Refinement on *F*^2^	Primary atom site location: structure-invariant direct methods
Least-squares matrix: full	Secondary atom site location: difference Fourier map
*R*[*F*^2^ > 2σ(*F*^2^)] = 0.036	Hydrogen site location: inferred from neighbouring sites
*wR*(*F*^2^) = 0.127	H-atom parameters constrained
*S* = 1.03	*w* = 1/[σ^2^(*F*_o_^2^) + (0.0881*P*)^2^ + 0.020*P*] where *P* = (*F*_o_^2^ + 2*F*_c_^2^)/3
2971 reflections	(Δ/σ)_max_ = 0.001
245 parameters	Δρ_max_ = 0.50 e Å^−^^3^
0 restraints	Δρ_min_ = −0.50 e Å^−^^3^

### Special details

**Table:**

Geometry. All e.s.d.'s (except the e.s.d. in the dihedral angle between two l.s. planes) are estimated using the full covariance matrix. The cell e.s.d.'s are taken into account individually in the estimation of e.s.d.'s in distances, angles and torsion angles; correlations between e.s.d.'s in cell parameters are only used when they are defined by crystal symmetry. An approximate (isotropic) treatment of cell e.s.d.'s is used for estimating e.s.d.'s involving l.s. planes.
Refinement. Refinement of *F*^2^ against ALL reflections. The weighted *R*-factor *wR* and goodness of fit *S* are based on *F*^2^, conventional *R*-factors *R* are based on *F*, with *F* set to zero for negative *F*^2^. The threshold expression of *F*^2^ > σ(*F*^2^) is used only for calculating *R*-factors(gt) *etc*. and is not relevant to the choice of reflections for refinement. *R*-factors based on *F*^2^ are statistically about twice as large as those based on *F*, and *R*- factors based on ALL data will be even larger.

### Fractional atomic coordinates and isotropic or equivalent isotropic displacement parameters (Å^2^)

**Table:**

	*x*	*y*	*z*	*U*_iso_*/*U*_eq_	
Cu1	1.25554 (4)	0.334248 (14)	0.91097 (6)	0.03596 (18)	
N2	1.0793 (3)	0.30297 (10)	0.7883 (4)	0.0331 (6)	
O1	0.8837 (2)	0.42750 (9)	1.2312 (4)	0.0429 (6)	
H1A	0.8138	0.4356	1.2531	0.064*	
O6	0.3423 (2)	0.55931 (9)	0.7333 (4)	0.0423 (6)	
H5WA	1.3391	0.4178	0.6721	0.063*	
H5WB	1.2197	0.4224	0.6553	0.063*	
N1	1.2308 (2)	0.40329 (10)	1.0943 (4)	0.0329 (6)	
O3	1.5049 (2)	0.53445 (8)	1.2189 (4)	0.0404 (6)	
O2	0.9233 (2)	0.52059 (8)	1.2933 (3)	0.0372 (5)	
O4	1.3867 (2)	0.58268 (9)	1.3819 (4)	0.0455 (6)	
C8	1.0490 (3)	0.24710 (12)	0.7920 (5)	0.0347 (7)	
H8	1.1159	0.2213	0.8425	0.042*	
C10	0.8215 (3)	0.26339 (11)	0.6516 (4)	0.0267 (6)	
C6	0.9569 (3)	0.47324 (12)	1.2534 (4)	0.0296 (7)	
C16	0.5797 (3)	0.27900 (12)	0.5599 (4)	0.0308 (7)	
H16	0.5926	0.3183	0.5789	0.037*	
O5	1.2742 (2)	0.39559 (9)	0.6563 (4)	0.0489 (6)	
H6WA	0.3745	0.5267	0.7734	0.073*	
H6WB	0.3550	0.5631	0.6190	0.073*	
C3	1.1825 (3)	0.50477 (11)	1.2678 (4)	0.0284 (7)	
H3	1.1658	0.5389	1.3253	0.034*	
C2	1.0874 (3)	0.46268 (11)	1.2204 (4)	0.0276 (6)	
C4	1.3011 (3)	0.49604 (11)	1.2300 (4)	0.0295 (7)	
C11	0.8532 (3)	0.32095 (12)	0.6460 (5)	0.0341 (7)	
H11	0.7882	0.3477	0.5968	0.041*	
C9	0.9247 (3)	0.22588 (12)	0.7254 (4)	0.0314 (7)	
H9	0.9095	0.1867	0.7295	0.038*	
C13	0.6855 (3)	0.24224 (11)	0.5851 (4)	0.0269 (6)	
C7	1.4074 (3)	0.54121 (11)	1.2813 (5)	0.0311 (7)	
C14	0.6585 (3)	0.18475 (12)	0.5473 (5)	0.0312 (7)	
H14	0.7261	0.1586	0.5593	0.037*	
C12	0.9796 (3)	0.33862 (12)	0.7125 (5)	0.0366 (8)	
H12	0.9975	0.3775	0.7049	0.044*	
C5	1.3199 (3)	0.44466 (12)	1.1400 (5)	0.0338 (7)	
H5	1.3991	0.4389	1.1101	0.041*	
C1	1.1176 (3)	0.41237 (12)	1.1376 (4)	0.0306 (7)	
H1	1.0556	0.3833	1.1106	0.037*	
C17	0.4562 (3)	0.25688 (12)	0.5068 (5)	0.0337 (7)	
H17	0.3866	0.2820	0.4929	0.040*	
N3	0.4300 (2)	0.20061 (10)	0.4737 (4)	0.0304 (6)	
C15	0.5326 (3)	0.16626 (11)	0.4921 (5)	0.0334 (7)	
H15	0.5175	0.1274	0.4659	0.040*	

### Atomic displacement parameters (Å^2^)

**Table:**

	*U*^11^	*U*^22^	*U*^33^	*U*^12^	*U*^13^	*U*^23^
Cu1	0.0197 (3)	0.0308 (3)	0.0561 (3)	0.00068 (14)	0.0077 (2)	−0.00424 (16)
N2	0.0244 (15)	0.0280 (12)	0.0466 (15)	−0.0045 (11)	0.0086 (12)	−0.0057 (11)
O1	0.0288 (14)	0.0324 (11)	0.0746 (16)	−0.0040 (10)	0.0262 (13)	−0.0099 (11)
O6	0.0284 (14)	0.0416 (12)	0.0603 (15)	0.0022 (10)	0.0178 (11)	−0.0056 (11)
N1	0.0236 (15)	0.0269 (12)	0.0500 (16)	−0.0006 (10)	0.0130 (13)	−0.0076 (10)
O3	0.0279 (13)	0.0320 (11)	0.0676 (16)	−0.0059 (9)	0.0235 (12)	−0.0052 (10)
O2	0.0320 (13)	0.0281 (11)	0.0554 (14)	0.0056 (9)	0.0184 (11)	−0.0027 (9)
O4	0.0433 (15)	0.0270 (11)	0.0730 (16)	−0.0090 (10)	0.0275 (13)	−0.0154 (11)
C8	0.0261 (19)	0.0266 (14)	0.0503 (19)	0.0014 (13)	0.0081 (15)	−0.0025 (13)
C10	0.0253 (18)	0.0255 (13)	0.0311 (15)	−0.0036 (12)	0.0103 (13)	−0.0030 (11)
C6	0.0250 (17)	0.0289 (14)	0.0357 (16)	0.0007 (12)	0.0090 (13)	0.0027 (12)
C16	0.0269 (18)	0.0219 (13)	0.0443 (17)	−0.0018 (12)	0.0109 (14)	−0.0043 (12)
O5	0.0370 (15)	0.0440 (13)	0.0656 (16)	−0.0032 (11)	0.0133 (12)	0.0091 (11)
C3	0.0277 (17)	0.0222 (13)	0.0359 (16)	0.0019 (12)	0.0092 (14)	−0.0021 (11)
C2	0.0255 (17)	0.0247 (13)	0.0344 (15)	0.0016 (12)	0.0109 (13)	0.0021 (11)
C4	0.0255 (18)	0.0237 (13)	0.0414 (17)	−0.0009 (12)	0.0125 (14)	0.0017 (12)
C11	0.0249 (18)	0.0242 (13)	0.0525 (19)	0.0017 (13)	0.0087 (15)	−0.0008 (13)
C9	0.0273 (18)	0.0225 (13)	0.0453 (17)	−0.0029 (12)	0.0113 (14)	−0.0013 (12)
C13	0.0234 (17)	0.0285 (14)	0.0287 (14)	−0.0037 (12)	0.0065 (12)	0.0001 (11)
C7	0.0274 (18)	0.0204 (13)	0.0451 (18)	−0.0005 (12)	0.0086 (15)	0.0046 (12)
C14	0.0209 (17)	0.0245 (13)	0.0472 (18)	0.0003 (12)	0.0070 (14)	−0.0010 (13)
C12	0.0254 (19)	0.0245 (14)	0.058 (2)	−0.0048 (12)	0.0071 (16)	−0.0021 (13)
C5	0.0257 (18)	0.0311 (15)	0.0477 (19)	0.0005 (13)	0.0151 (15)	−0.0070 (13)
C1	0.0258 (17)	0.0275 (14)	0.0387 (16)	−0.0029 (13)	0.0090 (14)	−0.0026 (12)
C17	0.0259 (18)	0.0246 (13)	0.0502 (19)	0.0005 (13)	0.0090 (15)	−0.0020 (13)
N3	0.0214 (14)	0.0297 (12)	0.0389 (14)	−0.0037 (11)	0.0059 (11)	0.0004 (10)
C15	0.0285 (19)	0.0228 (14)	0.0484 (19)	−0.0028 (12)	0.0094 (15)	−0.0008 (12)

### Geometric parameters (Å, °)

**Table:**

Cu1—N3^i^	1.971 (2)	C16—H16	0.9300
Cu1—N2	1.989 (3)	O5—H5WA	0.8480
Cu1—N1	2.119 (2)	O5—H5WB	0.8518
Cu1—O5	2.336 (2)	C3—C4	1.372 (4)
N2—C8	1.344 (4)	C3—C2	1.387 (4)
N2—C12	1.345 (4)	C3—H3	0.9300
O1—C6	1.307 (3)	C2—C1	1.383 (4)
O1—H1A	0.8200	C4—C5	1.393 (4)
O6—H6WA	0.8511	C4—C7	1.520 (4)
O6—H6WB	0.8511	C11—C12	1.369 (5)
N1—C5	1.333 (4)	C11—H11	0.9300
N1—C1	1.334 (4)	C9—H9	0.9300
O3—C7	1.237 (4)	C13—C14	1.383 (4)
O2—C6	1.216 (3)	C14—C15	1.365 (4)
O4—C7	1.250 (4)	C14—H14	0.9300
C8—C9	1.377 (4)	C12—H12	0.9300
C8—H8	0.9300	C5—H5	0.9300
C10—C11	1.387 (4)	C1—H1	0.9300
C10—C9	1.395 (4)	C17—N3	1.349 (4)
C10—C13	1.486 (4)	C17—H17	0.9300
C6—C2	1.488 (4)	N3—C15	1.334 (4)
C16—C17	1.372 (4)	N3—Cu1^ii^	1.971 (2)
C16—C13	1.390 (4)	C15—H15	0.9300
			
N3^i^—Cu1—N2	132.42 (10)	C3—C4—C7	121.2 (2)
N3^i^—Cu1—N1	115.90 (10)	C5—C4—C7	121.1 (3)
N2—Cu1—N1	106.79 (10)	C12—C11—C10	120.5 (3)
N3^i^—Cu1—O5	99.25 (9)	C12—C11—H11	119.8
N2—Cu1—O5	98.75 (10)	C10—C11—H11	119.8
N1—Cu1—O5	92.69 (9)	C8—C9—C10	119.7 (3)
C8—N2—C12	116.0 (3)	C8—C9—H9	120.1
C8—N2—Cu1	123.5 (2)	C10—C9—H9	120.1
C12—N2—Cu1	120.25 (19)	C14—C13—C16	116.9 (3)
C6—O1—H1A	109.5	C14—C13—C10	121.3 (3)
H6WA—O6—H6WB	104.8	C16—C13—C10	121.8 (2)
C5—N1—C1	117.4 (2)	O3—C7—O4	125.9 (3)
C5—N1—Cu1	120.2 (2)	O3—C7—C4	118.1 (3)
C1—N1—Cu1	121.37 (19)	O4—C7—C4	116.1 (3)
N2—C8—C9	123.8 (3)	C15—C14—C13	120.0 (3)
N2—C8—H8	118.1	C15—C14—H14	120.0
C9—C8—H8	118.1	C13—C14—H14	120.0
C11—C10—C9	116.3 (3)	N2—C12—C11	123.7 (3)
C11—C10—C13	122.5 (3)	N2—C12—H12	118.2
C9—C10—C13	121.2 (2)	C11—C12—H12	118.2
O2—C6—O1	124.5 (3)	N1—C5—C4	123.6 (3)
O2—C6—C2	122.0 (3)	N1—C5—H5	118.2
O1—C6—C2	113.6 (2)	C4—C5—H5	118.2
C17—C16—C13	119.4 (3)	N1—C1—C2	123.5 (3)
C17—C16—H16	120.3	N1—C1—H1	118.3
C13—C16—H16	120.3	C2—C1—H1	118.3
Cu1—O5—H5WA	120.2	N3—C17—C16	123.6 (3)
Cu1—O5—H5WB	105.2	N3—C17—H17	118.2
H5WA—O5—H5WB	94.7	C16—C17—H17	118.2
C4—C3—C2	120.0 (3)	C15—N3—C17	116.1 (3)
C4—C3—H3	120.0	C15—N3—Cu1^ii^	118.36 (19)
C2—C3—H3	120.0	C17—N3—Cu1^ii^	125.5 (2)
C1—C2—C3	117.8 (3)	N3—C15—C14	123.9 (3)
C1—C2—C6	122.2 (3)	N3—C15—H15	118.1
C3—C2—C6	119.9 (2)	C14—C15—H15	118.1
C3—C4—C5	117.6 (3)		
			
N3^i^—Cu1—N2—C8	24.8 (3)	C17—C16—C13—C10	−177.1 (3)
N1—Cu1—N2—C8	−128.5 (3)	C11—C10—C13—C14	164.4 (3)
O5—Cu1—N2—C8	136.0 (3)	C9—C10—C13—C14	−16.3 (4)
N3^i^—Cu1—N2—C12	−161.7 (2)	C11—C10—C13—C16	−16.3 (5)
N1—Cu1—N2—C12	44.9 (3)	C9—C10—C13—C16	163.1 (3)
O5—Cu1—N2—C12	−50.6 (3)	C3—C4—C7—O3	−170.0 (3)
N3^i^—Cu1—N1—C5	45.4 (3)	C5—C4—C7—O3	9.0 (4)
N2—Cu1—N1—C5	−156.2 (2)	C3—C4—C7—O4	9.0 (4)
O5—Cu1—N1—C5	−56.3 (2)	C5—C4—C7—O4	−172.0 (3)
N3^i^—Cu1—N1—C1	−146.8 (2)	C16—C13—C14—C15	−1.4 (4)
N2—Cu1—N1—C1	11.6 (3)	C10—C13—C14—C15	178.0 (3)
O5—Cu1—N1—C1	111.6 (2)	C8—N2—C12—C11	1.5 (5)
C12—N2—C8—C9	−0.5 (5)	Cu1—N2—C12—C11	−172.4 (3)
Cu1—N2—C8—C9	173.2 (2)	C10—C11—C12—N2	−0.9 (5)
C4—C3—C2—C1	0.8 (4)	C1—N1—C5—C4	0.2 (4)
C4—C3—C2—C6	−177.3 (3)	Cu1—N1—C5—C4	168.5 (2)
O2—C6—C2—C1	−166.9 (3)	C3—C4—C5—N1	−1.9 (5)
O1—C6—C2—C1	11.8 (4)	C7—C4—C5—N1	179.0 (3)
O2—C6—C2—C3	11.2 (4)	C5—N1—C1—C2	2.2 (4)
O1—C6—C2—C3	−170.2 (3)	Cu1—N1—C1—C2	−166.0 (2)
C2—C3—C4—C5	1.3 (4)	C3—C2—C1—N1	−2.7 (4)
C2—C3—C4—C7	−179.7 (3)	C6—C2—C1—N1	175.4 (3)
C9—C10—C11—C12	−0.7 (5)	C13—C16—C17—N3	−1.1 (5)
C13—C10—C11—C12	178.7 (3)	C16—C17—N3—C15	−1.1 (5)
N2—C8—C9—C10	−1.1 (5)	C16—C17—N3—Cu1^ii^	176.6 (2)
C11—C10—C9—C8	1.6 (4)	C17—N3—C15—C14	2.1 (5)
C13—C10—C9—C8	−177.8 (3)	Cu1^ii^—N3—C15—C14	−175.7 (3)
C17—C16—C13—C14	2.3 (4)	C13—C14—C15—N3	−0.9 (5)

Symmetry codes: (i) *x*+1, −*y*+1/2, *z*+1/2; (ii) *x*−1, −*y*+1/2, *z*−1/2.

### Hydrogen-bond geometry (Å, °)

**Table:**

*D*—H···*A*	*D*—H	H···*A*	*D*···*A*	*D*—H···*A*
O1—H1A···O6^iii^	0.82	1.69	2.502 (3)	168.
O5—H5WB···O2^iv^	0.85	2.12	2.959 (3)	167.
O6—H6WA···O3^iv^	0.85	1.91	2.694 (3)	152.
O5—H5WA···O3^v^	0.85	1.98	2.809 (3)	165.
O6—H6WB···O4^vi^	0.85	1.84	2.682 (3)	171.

Symmetry codes: (iii) −*x*+1, −*y*+1, −*z*+2; (iv) −*x*+2, −*y*+1, −*z*+2; (v) −*x*+3, −*y*+1, −*z*+2; (vi) *x*−1, *y*, *z*−1.
